# Development of an Improved Method of Sample Extraction and Quantitation of Multi-Mycotoxin in Feed by LC-MS/MS

**DOI:** 10.3390/toxins12070462

**Published:** 2020-07-19

**Authors:** Bahar Nakhjavan, Nighat Sami Ahmed, Maryam Khosravifard

**Affiliations:** Center for Analytical Chemistry, California Department of Food and Agriculture, Sacramento, CA 95832, USA; Nighat.ahmed@cdfa.ca.gov (N.S.A.); Maryam.khosravifard@cdfa.ca.gov (M.K.)

**Keywords:** mycotoxins, feed, modified QuEChERS, LC-MS/MS

## Abstract

A multi-mycotoxin chromatographic method was developed and validated for the simultaneous quantitation of aflatoxins (AFB1, AFB2, AFG1 and AFG2), ochratoxin A (OTA), zearalenone (ZON), deoxynivalenol (DON), nivalenol (NIV), diacetoxyscirpenol (DAS), fumonisins (FB1, FB2 and FB3), T-2 toxin (T-2) and HT-2 toxin (HT-2) in feed. The three most popular sample preparation techniques for determination of mycotoxins have been evaluated, and the method with highest recoveries was selected and optimized. This modified QuEChERS (quick, easy, cheap, effective, rugged and safe) approach was based on the extraction with acetonitrile, salting-out and cleanup with lipid removal. A reconstitution process in methanol/water was used to improve the MS responses and then the extracts were analyzed by LC-MS/MS. In this method, the recovery range is 70–100% for DON, DAS, FB1, FB2, FB3, HT-2, T-2, OTA, ZON, AFG1, AFG2, AFB1 and AFB2 and 55% for NIV in the spike range of 2–80 µg/kg. Method robustness was determined with acceptable z-scores in proficiency tests and validation experiments.

## 1. Introduction

Mycotoxins are the most common contaminants in agricultural crops produced by several species of mold and fungi. During growth, maturity, harvest, storage and processing of food and animal feed products, the fungus produces mycotoxins and other secondary metabolites [1]. These mycotoxin-contaminated food and feed threaten human and animal health even at very low concentration [2]. Various degrees of toxicity in food commodities can cause acute or chronic diseases such as immune suppression, cancer, pathological lesions and growth problems [3,4]. Moreover, the presence of mycotoxins in consuming animal products such as milk and meat are a significant safety concern as well [5,6]. Hence, creating an accurate and fast analytical method to quantify the contamination levels of mycotoxins plays a vital role in food and feed safety assessment risks.

Among hundreds of mycotoxins, a few have been recognized as a food safety concern including aflatoxins, fumonisins, ochratoxin A, zearalenone, deoxynivalenol, nivalenol, diacetoxyscirpenol, T-2 and HT-2. After two decades of research, simultaneous quantitative determination of mycotoxins and their derivatives in one analysis is challenging due to the wide polarity, solubility and physicochemical properties of these compounds.

Food crops and feed materials can be easily exposed to moisture that is needed for the growth of molds and fungi. High level of contamination by these mycotoxins has been frequently reported in food and feed. This attracted much attention in recent years due to high risk of contamination and consumption of these commodities all over the world [7,8].

Among a wide variety of mycotoxin sample preparations, accelerated solvent extraction [9], ultrasonic extraction [10], liquid-liquid extraction [11], immunoaffinity column [12] and solid-phase extraction [13] have been intensively researched. Most of the existing methods suffer from poor recovery, insufficient sensitivity and non-reproducibility. This makes these methods unsuitable for simultaneous determination of multi-mycotoxin. Chemical diversity, polarity and solubility of the mycotoxins are important characteristics that can significantly affect extraction efficiency.

Despite all these significant features, co-eluting of matrix components is the most challenging issue. Co-eluting matrix components creates enhancement or suppression of an analyte ionization affecting quantitation [14,15]. Matrix-matched calibration, standard addition and isotopic internal standards are the common solutions for compensation of this problem [16]. Matrix-matched calibration standards were used to reduce the interferences from the extraction process and improve the quantitation results. Therefore, developing an extraction technique to overcome all these challenges is daunting.

A variety of analytical instrumentations have been reported for mycotoxins such as TLC, GC-MS, LC-MS, HPLC-FLD, HPLC-UV and LC-MS/MS [17,18,19,20,21]. Liquid chromatography coupled with triple mass spectroscopy is the most recognized analytical instrumentation for the wide range of chemical contaminants such as mycotoxins in agricultural commodities. LC-MS/MS is known as a sensitive, selective, specific and efficient technique because of its versatility and reliability [22].

The objective of this work was to develop a robust and reliable extraction and clean up technique for multi-mycotoxin in a wide range of agricultural commodities by using LC-MS/MS and validate it through different approaches. The results illustrated here show that this sample preparation method can be applied in many laboratories analyzing feed materials.

## 2. Results and Discussion

### 2.1. Optimization of Extraction Process

Multi-mycotoxin extractions were prepared by using three different sample preparation techniques in corn. Immunoaffinity (method A) [23], solid-phase extraction (method B) [24] and QuEChERS (quick, easy, cheap, effective, rugged and safe) (method C) [25,26] methods applied for this study are shown in Table 1. They were evaluated based on analyte recovery (Rec.) and relative standard deviation (RSD).

The number of mycotoxins giving acceptable recoveries is seven in method A, eight in method B and nine in method C with significant improvement for DAS, FB1 and OTA in respect to the total number of 14 analyzed mycotoxins.

As shown in Table 1, extraction efficiency using acetonitrile-water in method C was increased for FB1 while there was no change for FB2 and FB3 due to their chemical structure. FB1 with two hydroxyl groups has more solubility than FB2 and FB3 with one hydroxyl group in acetonitrile-water as an extraction solution. Aqueous acetonitrile in method C in comparison with aqueous methanol and acetonitrile:methanol in methods A and B provided better recovery for FB1, DAS and OTA. In method C, cleaning of the sample extract was carried out by the EMR lipid removal in order to minimize ion source contamination. Thus, the QuEChERS method was further modified and used as a reference method. In method D, the extraction efficiency is affected by changing the pH because the addition of 0.3% formic acid in the extraction mixture changes the state of ionization for FB1, FB2, FB3, DAS and OTA as acidic and NIV as high polar compounds. It promotes the extraction of the neutral form of acidic mycotoxins into the organic phase due to their ionization constant. Moreover, this degree of acidity prevents the retention of acidic mycotoxins such as OTA in the cleanup process. Acetonitrile and methanol cannot extract NIV lonely, and the presence of low amounts of acid is necessary during extraction due to the incomplete partitioning. However, nivalenol as the most polar mycotoxin cannot meet the satisfactory level of recovery, 55% is a remarkable change in this report. The reconstitution step at the end of the process plays an important role in improving the signal responses and better peak shapes.

**Figure 1:**
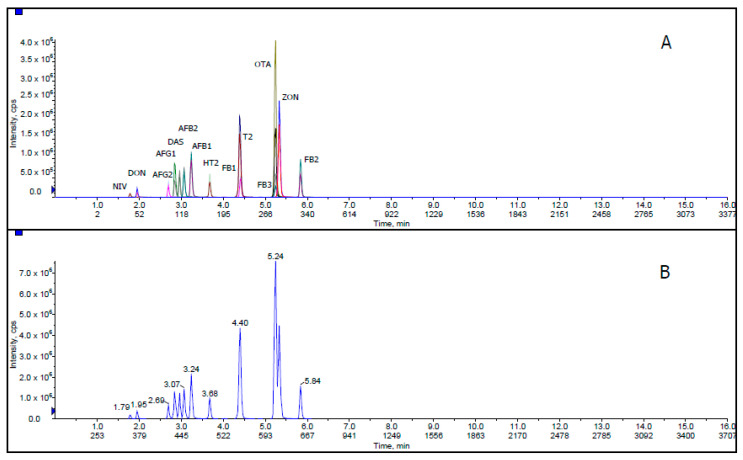
(**A**) Extracted ion chromatogram (XIC) and (**B**) total ion chromatogram (TIC) of mycotoxins at 50 ng/mL AFB1, AFB2, AFG1 and AFG2; 1000 ng/mL FB1, FB2, FB3, T-2, HT-2, DON and ZON; 2000 ng/mL NIV; 500 ng/mL DAS and OTA in corn matrix blank.

Figure 1 shows extracted ion chromatogram (XIC) and total ion chromatogram (TIC) for all mycotoxins with 50 ng/mL AFB1, AFB2, AFG1 and AFG2; 1000 ng/mL FB1, FB2, FB3, T-2, HT-2, DON and ZON; 2000 ng/mL NIV; 500 ng/mL DAS and OTA in corn matrix blank. XIC is a chromatogram created by taking intensity values at a single, discrete mass value, or a mass range, from a series of mass spectral scans. TIC is a chromatogram created by summing up intensities of all mass spectral peaks belonging to the same scan. The co-elution of some mycotoxin compounds was acceptable because these related compounds illustrate different MRM transitions in LC-MS/MS.

#### Solvent Mixture and Mobile Phase

Among different solvents, acetonitrile provides better recovery for a wide range of mycotoxins and matrices. However, there are reports of using methanol as a suitable co-extraction solvent in this field [27]. Different proportions of acetonitrile, methanol and water have been studied to discover an efficient extraction procedure with high recoveries for all analytes. In addition, to obtain the best chromatogram with the lowest noise signal, different mobile phases were used.

To optimize the extraction step, different proportions of acetonitrile and water were applied. Three volume ratios, acetonitrile:water (80:20, v:v), (60:40, v;v) and (50:50, v:v) were employed and acetonitrile:water (50:50, v:v) was confirmed as the most appropriate volume ratio. Various proportions of acidic water (0.1%, 0.2%, 0.3%, 0.5% and 1%) were tested and the extraction with equal proportion of acetonitrile and water containing 0.3% formic acid was suitable for all acidic and neutral mycotoxins.

Different various mobile phases and additives were evaluated, including acetonitrile and methanol (mobile phase) and formic acid, ammonium formate, ammonium fluoride, acetic acid and ammonium acetate (additive). Methanol containing formic acid, ammonium formate, and ammonium fluoride was found to give superior peak shape compared to acetonitrile. The reconstitution mixture was added at the end of the sample preparation step because the compatibility of mobile phases with final extraction solvent leads to improved signal response and prevents peak fronting and unstable retention time compared with no reconstitution. Since the nature of polar compounds makes them prone to dissolve in more polar solutions, reconstitution of samples with polar mobile phases is strongly recommended.

In this research, the combination of acetonitrile and acidic water as an extraction solvent at the beginning of the sample preparation and reconstitution with methanol:water (50:50, v:v) provides higher recoveries and better resolution for all 14 mycotoxins.

### 2.2. Validation

Animal feeds contain a mixture of crop ingredients, which makes them complex for mycotoxin analysis. Among the feedstuffs, corn has attracted the most attention in mycotoxin analysis due to its high production, consumption and contamination. This protein rich grain expedites the animal growth to prepare them for market weight quickly at a low cost. Corn is a good source of proteins, carbohydrates, vitamins, unsaturated lipids and minerals. It has a high level of matrix effect that makes it a great candidate for research. Therefore, the proposed extraction method was initially developed and validated in corn and further evaluated using different feed matrices.

**Figure 2:**
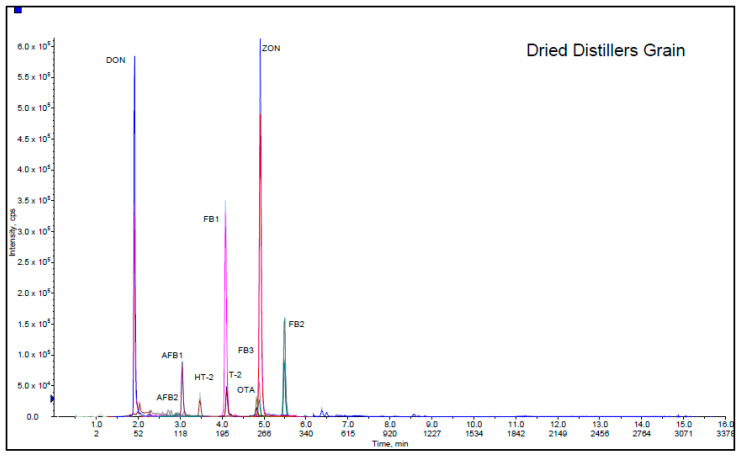
Extracted ion chromatogram of dried distillers’ grain.

As a part of validation study, this method was used in 5 different feed matrices (sheep food, dried distiller grain, dairy food, fish food and goat starter) provided from Association of American Feed Control Officials. AAFCO program materials were prepared from different commercial feedstuffs purchased from US marketplaces in order to monitor the use and performance of methods in analytical laboratories. Participating in these proficiency tests is a valid source of method evaluation because several laboratories take part simultaneously and determine 12 mycotoxins in various animal feeds with different level of contaminations. These samples are delivered to the laboratories as powdered samples, and there is not usually enough information about their ingredients and components. They are mixtures of various crops and agricultural products. The matrices considered in this validation were selected among 2019–2010 AAFCO proficiency tests. Sheep food, dried distiller grain, dairy food, fish food and goat starter represent a wide range of feedstuffs with diverse physicochemical properties. As shown in Table 2, the applicability of the method was confirmed by satisfactory results for all mycotoxins (z ≤ ±2), although it is worth noting that sheep food, dried distillers’ grain and fish food with lower z-scores were affected by the presence of co-eluting matrix interferences leading to signal suppression. The Chromatogram of the dried distiller grain is presented in Figure 2. It depicts the separation of DON, AFB2, AFB1, HT-2, FB1, T-2, FB3, OTA, ZON and FB2 at different retention times.

In addition, a FERN comparison exercise was conducted for Aflatoxin B1 in 12 dog foods, which was successfully reported. All these matrices were spiked and analyzed for quality control purpose.

Researches indicate that matrix effect is the most important factor in the extraction process for mycotoxin analysis in feed. To eliminate matrix effects, matrix-matched calibration must be in the same or similar matrices that are being studied. Therefore, it is possible that the matrix-matched calibration cannot completely account for different degrees of incurred mycotoxins, and matrix interferences lead to signal suppression and enhancement. As shown in the accuracy and precision section, the recoveries for the in-house validation were obtained using the same matrices for calibration standard and matrix spike samples while there was no such condition for samples in proficiency tests. Recoveries higher than 70% were obtained for DON, DAS, FB1, FB2, FB3, HT-2, T-2, OTA, ZON, AFG1, AFG2, AFB1 and AFB2 and 55% for NIV with relative standard deviation lesser than 12%.

The presented analytical procedure was used for the analysis of more than 50 routine feed samples. The applicability of this method was confirmed by comparison with our old laboratory methods using water and acetonitrile/0.5% acetic acid for extraction followed by salting out reagent and hexane. Additionally, the method validation was performed with three different levels of spike and five replicates.

**Figure 3:**
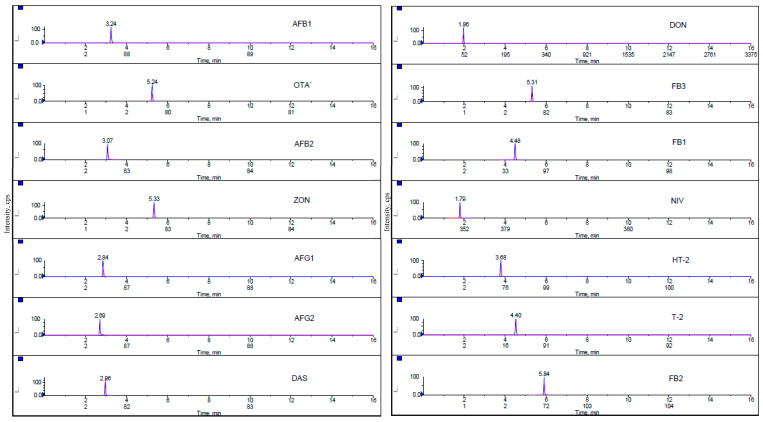
Multiple reaction monitoring (MRM) chromatograms of 14 mycotoxins at the lowest level of calibration standard.

Chromatograms of all analyzed mycotoxins in the lowest matrix-matched calibration standard are presented in Figure 3. Fragmentation reactions were carried out in Multiple Reaction Monitoring mode and two product ions, a quantifier ion and a qualifier ion, were measured for 14 mycotoxins. During the evaluation, it was proved that the sensitivity of the MRM transitions is related to the quality and freshness of the used solvents.

## 3. Conclusions

The wide range of agricultural products, contaminant ranges and different distribution ways make mycotoxin an important issue in the world. Concerns with potential human health impact associated with consumption of dairy products drive research in this field. Due to the complexity of animal feed products, developing a methodology for extraction and clean-up processes covering recognized mycotoxins is necessary. For this purpose, three analytical sample preparation techniques, immunoaffinity, solid-phase extraction and QuEChERS, were compared, and the best was optimized. Corn is considered to be a complex matrix with severe matrix interferences, and matrix-matched calibration was used to reduce ion source contamination and decrease matrix effect because of co-eluting matrix components. This LC-MS/MS method was designed to create a robust and reliable approach for simultaneous analysis of 14 mycotoxins in various feeds. These selected compounds with different degrees of toxicity are representative of an important group of mycotoxins. Recovery values ranged from 70–100% for DON, DAS, FB1, FB2, FB3, HT-2, T-2, OTA, ZON, AFG1, AFG2, AFB1 and AFB2 and 55% for NIV. The results from 5 AAFCO proficiency tests have been reported on various matrices along with in-house validation.

## 4. Materials and Methods

### 4.1. Chemicals and Reagents

HPLC grade acetonitrile, methanol, water and formic acid were purchased from Fisher Scientific. Japanese aflatoxin mixture (25 μg/mL) from Sigma-Aldrich; deoxynivalenol (25 µg/mL), nivalenol (25 µg/mL), zearalenone (10 µg/mL), ochratoxin A (10 µg/mL) from Romer Labs; diacetoxyscirpenol (100 µg/mL), T-2 toxin (100 µg/mL), HT-2 toxin (100 µg/mL), fumonisin B1 (100 µg/mL), fumonisin B2 (100 µg/mL), and fumonisin B3 (100 µg/mL) from Trilogy. QuEChERS EN extraction salts and Captiva EMR-Lipid cartridge were acquired from Agilent (CA, USA).

### 4.2. Sample Preservation and Storage

Samples were ground finely with an Ultra Centrifugal Retsch Mill ZM200 (Retsch GmbH, Haan, NRW., Germany) to pass a 500 µm sieve. After grinding, they were homogenized to a powder-like consistency. All samples were stored in freezer before and after extraction process. Each sample was mixed carefully before weighing.

### 4.3. Sample Preparation

Sample preparation and cleanup process are necessary steps in various matrices for mycotoxin determination. Without sample cleanup, analysis can damage the chromatography column and Mass spectrometer. Immunoaffinity (IMA), solid-phase extraction (SPE), QuEChERS and modified QuEChERS methods were described in this section.

#### 4.3.1. Immunoaffinity Column Method (Method A)

A corn sample (10 g) was extracted with 50 mL Phosphate Buffered Saline (PBS). After shaking for 60 min and centrifugation for 10 min, 35 mL of supernatant (extract A) was filtered through a glass microfiber filter. Subsequently, 35 mL methanol was added to the 15 mL remaining supernatant and the sample was shaken and centrifuged the same as extract A. After these two steps, 10 mL extract was diluted with 90 mL PBS and filtered through a glass microfiber filter (extract B). Extract B (50 mL) was passed over Myco6in1^+^ (multiantibody IMA column, VICAM, Watertown, MA, USA) and washed with 20 mL PBS. At this step, 5 mL extract A was passed through the same Myco6in1+ column and washed with 10 mL water. The extract was eluted two times with 1.5 mL methanol:water (80:20, v:v) containing 0.5% acetic acid. The last eluted step was repeated and collected in another vial. Then, the extract was evaporated to dryness at 40–50 °C under a gentle stream of nitrogen. Both tubes were reconstituted with methanol:water (80:20, v:v) containing 0.5% acetic acid and combined into one vial.

#### 4.3.2. Solid-Phase Extraction (Method B)

A corn sample (2 g) was weighed into a 50 mL centrifuge tube with 2 mL of water. 10 mL acetonitrile:water (50:50, v:v) was added and shaken for 15 min in Geno/Grinder (SPEX Sample Prep, Metuchen, NJ, USA). After centrifugation, the diluted supernatant was passed through the SPE cartridge (Oasis HLB, 500mg, 6 mL). Volumes of 3 mL water and 3 mL hexane were added into the cartridge and eluted by 2 mL acetonitrile and 2 mL methanol. The extract was evaporated and reconstituted with methanol:water (50:50, v:v).

#### 4.3.3. QuEChERS Method (Method C)

In a 50 mL centrifuge tube, 5 g of a corn sample was extracted with 10 mL of water and 10 mL of acetonitrile. After a brief vortex, the mix was placed in Geno/Grinder for 20 min to homogenize well. Anhydrous MgSO_4_ (4.0 g) and 0.5 g NaCl were added and shaken for 2 more minutes on a Geno/Grinder homogenizer and then centrifuged for 7 min. Subsequently, 1.6 mL of extract was diluted with 0.4 mL of water. The diluted sample was passed through a lipid removal column under constant vacuum and evaporated with nitrogen stream to the final volume of 1mL.

#### 4.3.4. Modified QuEChERS Method (method D)

A corn sample (5 g) was weighed into a 50 mL centrifuge tube with 10 mL of water containing 0.3% formic acid and 10 mL of acetonitrile. After a brief vortex, the mix was placed in Geno/Grinder for 20 min to homogenize well. QuEChERS salts were added and shaken for 2 more minutes on a Geno/Grinder homogenizer and then centrifuged for 7 min. Subsequently, 1.6 mL of the extract was diluted with 0.4 mL of water. The diluted sample was passed through a lipid removal under constant vacuum. The extract was evaporated to dryness at 40–50 °C under a gentle stream of nitrogen and reconstituted in mobile phase A/mobile phase B (50/50, v/v).

This modified QuEChERS method uses an acidic condition to increase the extraction efficiency by using water with 0.3% formic acid. The polarity of compounds can be changed by using different pH levels [28]. It also offers evaporation/reconstitution for improving the MS responses and peak broadening. However, nivalenol cannot be completely extracted in acetonitrile; acidic condition and compatibility with mobile phases are essential in this procedure. The presence of the 0.3% formic acid improves the analyte partitioning into the organic phase and its elution as the first compound in the chromatographic run. Moreover, it prevents the retention of Ochratoxin A on the lipid removal cartridge and provides better results. Finally, the salting-out step with anhydrous MgSO_4_ and NaCl followed by lipid removal and reconstitution steps achieved highest recoveries.

### 4.4. Matrix-Matched Calibration Preparation

All mycotoxin standard solutions were purchased from ISO 17034 accredited vendors. Aflatoxin mix (aflatoxin G1, G2, B1 and B2), and respectively, 25 µg/mL, DON 25 µg/mL, NIV 25 µg/mL, ZON 10 µg/mL, OTA 10 µg/mL, DAS 100 µg/mL, T-2 100 µg/mL, HT-2 100 µg/mL, FB1 100 µg/mL, FB2 100 µg/mL and FB3 100 µg/mL were used to prepare a combination standard. A mixture of 50 ng/mL AFB1, AFB2, AFG1 and AFG2; 1000 ng/mL FB1, FB2, FB3, T-2, HT-2, DON and ZON; 2000 ng/mL NIV, 500 ng/mL DAS and OTA in matrix blank was prepared for the highest level of calibration curve, and a mixture of 0.25 ng/mL AFB1, AFB2, AFG1 and AFG2; 5 ng/mL FB1, FB2, FB3, T-2, HT-2, DON and ZON; 10 ng/mL NIV, 2.5 DAS and OTA in matrix blank was prepared for the lowest level of calibration curve.

To create a matrix-matched calibration, eight point of standards must be prepared in the same or similar matrix group that was chosen for analysis. They were prepared in the same way as actual samples with 0.2% formic acid. All calibrant solutions for matrix-matched calibration were made by using clean matrix extracts. Mycotoxin standard solutions were stored in a refrigerator at 4 ± 4 °C.

### 4.5. Equipment Conditions

The liquid chromatography and MS/MS optimization were studied in this research to find the most appropriate operating conditions by individual injection of each mycotoxin standard.

#### 4.5.1. Liquid Chromatography Separation Conditions

Shimadzu liquid chromatography equipped with Kinetex C18 column (2.6 µm particle size, 100 × 3.00 mm, Phenomenex, Torrance, CA, USA) was used. The mobile phases were mobile phase A, 0.5 mM ammonium fluoride, 5 mM ammonium formate and 0.1% formic acid in water; mobile phase B, 0.5 mM ammonium fluoride, 5 mM ammonium formate and 0.1% formic acid in methanol. Samples were eluted using a gradient at a flow rate of 0.45 mL/min throughout the 16 min run-time at 40 °C with injection volume of 10 µL. The gradient conditions were optimized as follows: 10% B from 0.01 to 0.5 min, 10–50% B from 0.5 to 10 min, 100% from 10–11 min, 10% 11–13.5 and 0% B from 13.5 to 16 min.

#### 4.5.2. Mass Spectroscopy Conditions

To achieve a mass spectrum of the mycotoxins, a Triple Quad 5500 ABSciex mass spectrometer (AB SCIEX instruments, Foster, CA, USA) with a positive ESI interface is used. Mass spectrometer operating parameters are summarized as follows: curtain gas: 20 psig, ion spray voltage, 4500; temperature, 400 °C; ion source gas 1, 20; ion source gas 2, 30; collision gas, 8; MRM detection window, 60 sec and target scan time, 1. The mass spectrometer operates in scheduled MRM (Multiple Reaction Monitoring) mode described in Table 3, by monitoring 2 transitions and selecting the optimum voltage of declustering potential, collision energies and collision cell exit potentials for each compound. Sciex Analyst software 1.7 (AB SCIEX instruments, Foster, CA, USA) and Multiquant software 3.0 (AB SCIEX instruments, Foster, CA, USA) were applied for data acquisition and data processing respectively.

### 4.6. Method Validation

The proposed method was validated by an in-house quality control procedure. Instrumental linearity, method detection limit, reporting limit, accuracy and precision were estimated.

#### 4.6.1. Instrumental Linearity

A quadratic regression of the calibration data with all levels was used with weighted 1/x. Correlation coefficient (R^2^) was higher than 0.995 in all cases. The linearity was evaluated based on using eight-point calibration curves.

#### 4.6.2. Method Detection and Reporting Limit

Method detection limit (MDL) refers to the lowest concentration of the analyte that a method can detect reliably. To determine the MDL, 7 corn matrix blank samples were spiked at a different concentration for each analyte and processed through the entire method along with blank. The standard deviation derived from the spiked sample recoveries was used to calculate the MDL using this equation:

MDL = tS (*n* = 7 replicates, t = 3.143)

Reporting limit refers to a level at which reliable quantitative results may be obtained. The MDL is used as a guide to determine the RL. The RL is the two times the MDL in this work. The calculated MDL and RL for all mycotoxins are shown in Table 4.

#### 4.6.3. Accuracy and Precision

The method validation consisted of five sample sets and each set includes three levels of fortification. All spikes were processed through the entire analytical method. Spike levels, recoveries and standard deviation for the mycotoxins are shown in Table 5. The recoveries were within range of 70%–100% for 13 mycotoxins and 55% for NIV. The relative standard deviation of recoveries was lower than 12% for all mycotoxins.

## Figures and Tables

**Table 1:** Recovery (Rec.) and Relative Standard Deviation (RSD) values (*n* = 5) of 14 mycotoxins using different extraction techniques.

Analyte	Spike Level µg/kg	Method A Rec. (%) and RSD (%)	Method B Rec. (%) and RSD (%)	Method C Rec. (%) and RSD (%)	Method D Rec. (%) and RSD (%)
DON	40	94.4 and 7.1	100.2 and 8.4	83.6 and 1.9	85.0 and 4.2
DAS	20	10.6 and 9.9	12.1 and 18.5	82.6 and 5.5	96.1 and 3.7
FB1	40	31.7 and 19.6	38.9 and 19.9	62.8 and 6.3	75.7 and 4.2
FB2	40	55.5 and 9.5	58.5 and 9.0	54.7 and 3.0	78.9 and 5.3
FB3	40	58.7 and 7.7	60.5 and 4.5	67.6 and 7.1	76.9 and 4.7
HT-2	40	98.4 and 6.5	121.6 and 4.0	93.5 and 0.9	95.9 and 4.0
T-2	40	94.9 and 7.1	113.4 and 6.5	87.3 and 4.1	99.0 and 2.4
OTA	20	7.9 and 8.5	9.3 and 7.2	58.9 and 3.2	86.0 and 1.3
ZON	40	62.5 and 6.6	103.8 and 2.9	96.7 and 2.9	81.8 and 6.0
AFG1	2.0	108.4 and 3.5	109.5 and 4.2	87.5 and 4.2	82.9 and 2.8
AFG2	2.0	111.3 and 4.4	112.0 and 1.9	91.6 and 3.4	88.8 and 5.4
AFB1	2.0	81.8 and 4.3	86.7 and 6.6	78.5 and 3.4	79.2 and 0.94
AFB2	2.0	83.6 and 5.7	94.3 and 5.9	84.4 and 2.9	84.5 and 5.2
NIV	80	ND	ND	ND	58.8 and 3.7

Method A (Vicam), Method B (solid-phase extraction (SPE)), Method C (QuEChERS (quick, easy, cheap, effective, rugged and safe)) and Method D (Modified QuEChERS).

**Table 2:** Results of mycotoxin analysis in Association of American Feed Control Officials (AAFCO) proficiency tests.

Sample Type	Sheep Food			Dried Distillers Grain			Dairy Food			Fish Food			Goat Starter		
Toxin	Assessed Value, µg/kg	Lab Value, µg/kg	z-Score	Assessed Value, µg/kg	Lab Value, µg/kg	z-Score	Assessed Value, µg/kg	Lab Value, µg/kg	z-Score	Assessed Value, µg/kg	Lab Value, µg/kg	z-Score	Assessed Value, µg/kg	Lab Value, µg/kg	z-Score
Don	2181	993	−1.8	9673	4559	−1.8	5354	4036	−0.8	2366	988	−1.9	2221	1615	−0.91
FB1	4432	1770	−2.0	1306	560	−1.8	2905	2016	−1.0	2470	991	−2.0	3190	3321	0.14
FB2	1332	460	−2.1	373	155	−1.8	844	698	−0.6	581	233	−1.9	933	875	−0.20
FB3	595	234	−1.9	189	100	−1.4	357	380	0.2	337	164	−1.6	ND	ND	ND
HT-2	96.1	57.7	−1.2	76.0	58.4	−0.7	74	97	−1.0	37.2	27	−0.8	234	288	0.72
T-2	91.5	30.0	−2.0	55.0	25.5	−1.6	97	60.5	−1.2	69.5	28	−1.8	262	214	-0.57
OTA	107	63.8	1.2	7.6	6.3	−0.5	151	200	1.0	186.7	103	−1.4	316	364	0.48
ZON	335	126	−1.9	574	231.1	−1.9	885	730	−0.6	368.3	144	−1.9	377	342	-0.30
AFG1	0.59	0.62	0.14	ND	ND	ND	ND	ND	ND	ND	ND	ND	1.37	1.6	0.46
AFG2	ND	ND	ND	ND	ND	ND	ND	ND	ND	ND	ND	ND	ND	ND	ND
AFB1	24.3	14.1	−1.2	15.1	4.4	−2.1	38.5	28.4	−0.8	53.7	17.6	−2.0	85.8	89.5	0.13
AFB2	1.5	0.6	−1.7	1.3	1.0	−0.6	7.8	6.2	−0.6	3.5	1.1	−1.9	16	23	1.3

% satisfactory z-scores (z ≤ ±2), questionable z-scores (z ≤ ±3) and unsatisfactory z-scores (z ≥ ±3).

**Table 3:** MRM parameters for mycotoxin detection.

Analyte	Type	Q1 (m/z)	Q3 (m/z)	Retention Time (min)	DP (Volts)	CE (Volts)	CXP (Volts)
DON	[M+H]^+^	297.2	249.2 231.1	1.93	37	16 18	16 16
DAS	[M+H]^+^	384.0	307.1 105.1	3.00	54	9 40	27 20
FB1	[M+H]^+^	722.2	334.3 352.4	4.23	75	53 50	16 16
FB2	[M+H]^+^	706.1	336.3 318.4	5.67	73	48 50	14 14
FB3	[M+H]^+^	706.1	336.3 318.4	5.04	73	48 50	14 14
HT-2	[M+H]^+^	442.2	263.2 215.1	3.71	39	25 28	14 11
T-2	[M+H]^+^	484.2	215.2 185.1	4.42	38	29 38	15 16
OTA	[M+H]^+^	404.0	239.0 358.1	5.14	42	31 19	13 15
ZON	[M+H]^+^	319.2	283.2 187.1	5.26	60	17 26	15 15
AFG1	[M+H]^+^	329.0	243.1 283.1	2.78	73	37 35	16 16
AFG2	[M+H]^+^	331.1	245.1 257.1	2.64	70	41 42	16 16
AFB1	[M+H]^+^	313.1	285.1 241.1	3.17	58	32 51	14 18
AFB2	[M+H]^+^	315.2	287.2 259.1	2.99	43	36 41	12 17
NIV	[M+H]^+^	313.1	175.1 115.1	1.77	96	21 73	12 8

Q1: first quadrupole; Q3: third quadrupole; DP: declustering potential; CE: collision energy; CXP: collision cell exit potential.

**Table 4:** MDL Study for Mycotoxins in Corn (*n* = 7).

Toxin	Spike (µg/mL)	SD	MDL	RL
DON	0.08	0.00023	0.00713	0.01426
DAS	0.04	0.00115	0.00360	0.00720
FB1	0.24	0.01588	0.04991	0.09983
FB2	0.24	0.01357	0.04266	0.08533
FB3	0.08	0.00348	0.01094	0.02189
HT-2	0.08	0.00115	0.00362	0.00724
T-2	0.08	0.00122	0.00383	0.00766
OTA	0.04	0.00159	0.00498	0.00997
ZON	0.08	0.00269	0.00845	0.01690
AFG1	0.004	0.00010	0.00032	0.00064
AFG2	0.004	0.00016	0.00049	0.00098
AFB1	0.004	0.00012	0.00037	0.00073
AFB2	0.004	0.00006	0.00018	0.00035
NIV	0.16	0.00456	0.01433	0.02867

SD: Standard Deviation, MDL: Method Detection Limit and RL: Reporting Limit.

**Table 5:** Accuracy and precision of LC-MS/MS method for determination of 14 spiked Corn (*n* = 5).

Mycotoxins	Concentration (µg/g)	Mean Recovery (%)	SD (µg/g)	RSD (%)	Mycotoxins	Concentration (µg/g)	Mean Recovery (%)	SD (µg/g)	RSD (%)
DON	0.06	87.3	2.9	3.3	OTA	0.03	86.9	6.1	7.1
	0.08	86.1	2.1	2.4		0.04	89.0	89.0	4.3
	0.2	86.0	1.9	2.2		0.1	94.7	94.7	7.3
							
DAS	0.03	93.8	5.8	6.2	ZON	0.06	78.7	2.2	2.9
	0.04	96.1	7.6	7.9		0.08	81.9	5.7	7.0
	0.1	91.7	2.3	2.5		0.02	87.2	5.5	6.3
							
FB1	0.24	75.8	8.9	10.6	AFG1	0.003	84.7	10.5	12.3
	0.6	74.4	6.7	9.1		0.004	86.3	7.3	8.5
	2.4	80.4	1.7	2.1		0.01	81.9	6.1	7.4
							
FB2	0.24	71.3	5.3	8.7	AFG2	0.003	99.1	8.7	8.8
	0.6	74.0	2.9	4.0		0.004	98.0	4.9	5.0
	2.4	74.6	3.5	5.1		0.01	92.3	2.6	2.9
							
FB3	0.08	74.2	6.7	9.0	AFB1	0.003	79.6	5.7	7.2
	0.2	77.6	3.9	5.1		0.004	82.0	3.3	4.1
	0.8	78.2	4.6	5.9		0.01	79.3	3.5	4.4
							
HT-2	0.06	97.5	2.5	2.6	AFB2	0.003	86.1	6.2	7.2
	0.08	97.0	4.2	4.3		0.004	86.5	6.9	7.9
	0.5	84.1	4.4	5.2		0.01	81.9	4.4	5.3
							
T-2	0.06	96.1	3.5	3.6	NIV	0.12	55.0	3.7	6.7
	0.08	98.4	3.4	3.5		0.16	56.7	3.6	6.3
	0.2	98.4	4.3	4.4		0.4	55.0	4.1	7.5
							
